# Post-Polymerization Modification of Ring Opening Metathesis Polymerization (ROMP)-Derived Materials Using Wittig Reactions

**DOI:** 10.3390/polym12061247

**Published:** 2020-05-29

**Authors:** Ryan Duty, Christopher E. Hobbs

**Affiliations:** Department of Chemistry, Sam Houston State University, Huntsville, TX 77347, USA; rsd015@shsu.edu

**Keywords:** wittig reaction, ROMP, post-polymerization modification

## Abstract

This communication describes our recent efforts to utilize Wittig olefination reactions for the post-polymerization modification of polynorbornene derivatives prepared through ring opening metathesis polymerization (ROMP). Polymerizing α-bromo ester-containing norbornenes provides polymers that can undergo facile substitution with triphenylphosphine. The resulting polymeric phosphonium salt is then deprotonated to form an ylide that undergoes reaction with various aryl aldehydes in a one-pot fashion to yield the respective cinnamates. These materials can undergo further modification through photo-induced [2 + 2] cycloaddition cross-linking reactions.

## 1. Introduction

Post-polymerization modifications (PPMs) offer chemists the ability to change or fine-tune a macromolecule’s overall bulk properties. These reactions are typically carried out through the covalent modification of a polymer’s end groups, backbone, and/or pendant groups [1]. Although Hermann Staudinger receives much credit (deservedly so) for the development of “polymer-analogous reactions”, these types of transformations have been carried out for much longer [2]. However, recent decades have been witness to an explosion of literature examples describing various types of PPM reactions [3,4,5], including the modification of bio-based [6] polymers and plastic upcycling [7,8]. However, this renaissance is mostly attributable to the invention and development of the concepts of “click” and “green” chemistry [9,10], as well as controlled [11] “living” polymerization techniques, such as reversible addition/fragmentation chain transfer (RAFT) polymerization and atom transfer radical polymerization (ATRP) [12,13]. For example, Zhao et al. have recently shown that polymers containing Meldrum’s acid derivatives (prepared using RAFT) could undergo post-polymerization Knoevenagel reactions [14], while Sumerlin and coworkers showed that keto-enol tautomerization could be harnessed for the PPM of materials prepared from RAFT [15]. Bode’s laboratory recently showed that it was possible to utilize ATRP to prepare acyltrifluoroborate-containing polymers that could undergo post-synthetic modifications with functionalized amines [16].

In addition to RAFT and ATRP, ring opening metathesis polymerization (ROMP [17]) has become one of the most widely utilized living polymerization techniques. This is thanks, in part, to the high functional group tolerance (and bench-stability) of Grubbs-type initiators that can easily facilitate the polymerization of high- and medium-strain cyclic monomers (i.e., norbornenes, cyclooctenes, etc.) for a number of applications [18]. Despite the high functional group tolerance of Grubbs initiators, there exist circumstances in which PPM reactions are required. For example, Nelson and coworkers recently described the use of Dewar heterocycles as monomers for ROMP [19]. Such species could be polymerized to provide polymeric -lactams that could undergo subsequent modifications to yield water-soluble -amino acid polymers, functionalities that may be less well- tolerated in ROMP. Kiessling’s laboratory reported on the ROMP of oxazinone-based monomers. The resulting poly(oxazinone)s could undergo PPM with a variety of oxime ethers [20]. This same laboratory pioneered the use of “activated” *N*-hydroxysuccinimide (NHS) ester-containing ROMP materials, that could undergo PPMs to form biologically-active polymers [21]. Our lab has recently adopted this technique for the preparation of a polymer-supported DMAP catalyst [22]. Earlier examples describing the utility of multiple “click” reactions as tools for PPM of ROMP-based materials exist, as do those detailing PPM strategies to prepare polymer/protein conjugates [23,24,25,26,27]. 

For the last few years, our laboratory [28,29,30,31,32,33] (among others [34,35,36]) has been interested in the utilization of a “thio-bromo” click [37,38] reaction for carrying out PPMs. Of particular interest to us is the modification of polymeric materials prepared from ROMP [21]. The marriage of these two chemistries required the polymerization of norbornene/cyclooctene derivatives-decorated with electrophilic α-bromo ester moieties that could undergo PPMs through reactions with nucleophilic thiols. The utility of pendant alkyl bromides has been illustrated for other substitution reactions as well [39,40,41,42,43,44,45]. Following our initial reports, we reasoned that this functionality may serve as a convenient handle to introduce other useful functional groups, such as acrylates and cinnamates; these are α,β-unsaturated carbonyls that can undergo further modifications through various Michael reactions, as well as photo-facilitated [2 + 2] cycloadditions. Polymers decorated with acrylates and cinnamates have indeed been prepared through ROMP [46,47,48,49]. However, most of these rely on multistep monomer syntheses (≥ 2 steps) utilizing standard, substitution chemistry in a pre-polymerization strategy. 

To this day, the Wittig reaction remains a highly efficient route toward the preparation of alkenes, including acrylate and cinnamate esters [50]. For this reason, it is a mainstay in sophomore organic chemistry courses. Although the Wittig and Wittig-like reactions have been used to prepare polymers and modify the termini of ROMP-based materials [51], it has not, to our knowledge, been utilized as PPM reaction for the modification of polymer-pendant groups, especially not with ROMP. The reason for its underutilization is, more-than-likely, the lack of atom-economy, a hallmark of “green chemistry”. This low atom economy is a consequence of the formation of a stoichiometric equivalent of a triphenylphospine oxide which is, by no means, benign. For this reason, this reaction is often thought to be antithetical to the green chemistry philosophy (although catalytic variants have been developed [52]). Product purification only exacerbates this issue, as the removal of the byproduct often requires column chromatography, generating even more waste. However, this actually may not be as problematic for modifying polymers (especially on academic-lab scales), since polymer purification is commonly carried out by solvent precipitation. While not inherently “green”, solvent precipitations are relatively green, when compared to column chromatography (which can generate excessive amounts of solvent waste). Furthermore, this can offer a simpler way to isolate the phosphine oxide byproduct, which could presumably be reduced and recycled. With this in mind, we developed a straightforward method for modifying poly(norbornene) derivatives using Wittig reactions.

## 2. Results and Discussion

**Scheme 1:**
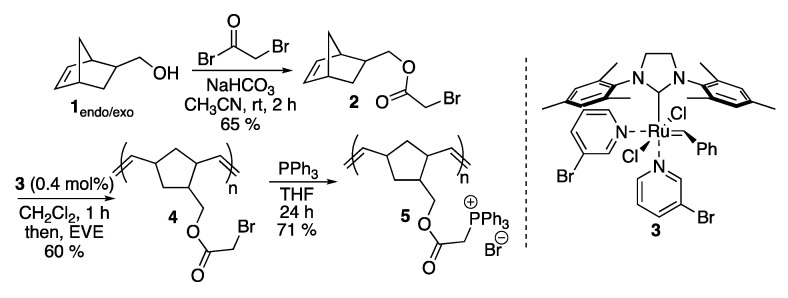
Synthesis of phosphonium salt **5**.

Our work began with the preparation of α-bromo ester-decorated norbornene **2** using procedures adapted from the literature [53]. This involved subjecting commercially available alcohol (mixture of endo and exo) to a reaction with bromoacetyl bromide in the presence of sodium bicarbonate for 2 h (Scheme 1). This led to the isolation of **2** in 65% yield. **2** was then subjected to ROMP in the presence of Grubbs 3rd generation initiator **3** [54] using [M]:[I] ratio of 250:1 in methylene chloride for 2 h. Subsequent quenching with ethyl vinyl ether (EVE) and precipitation into methanol led to the isolation of polymer **4** as gummy solid in 80% yield. Product formation was confirmed by the disappearance of the olefinic signals between 6.2 and 5.9 ppm and the appearance of broad signals between 5.48 and 5.07 ppm in the ^1^H NMR spectrum. Gel permeation chromatography (GPC) revealed that **4** possesses *M_n_* and *Ð* values of 64,500 Da (*M_n,theor._* = 61,500 Da) and 1.03, respectively (see details of ^1^H NMR spectra and GPC chromatograms in Supplementary Materials). 

**Scheme 2:**
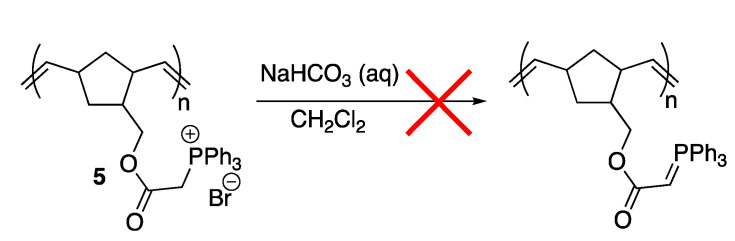
Attempted synthesis of polymeric phosphorus ylide from **5**.

In order to prepare the requisite polymeric ylide, we next subjected polymer **4** to a substitution reaction with triphenylphosphine in THF for 24 h. Phosphonium salt **5** was isolated in 71% yield by solvent precipitation into ether. Confirmation was obtained by ^1^H NMR, which showed a disappearance of the broad singlet at 3.85 ppm, corresponding to the α-protons geminal to the bromide; this signal was shifted upfield and overlapped with the polymer’s olefinic signals. Phosphonium salt **5** was next subjected to reaction with sodium bicarbonate to generate a polymeric ylide (Scheme 2). Unfortunately, we found this ylide formation to be problematic, since it led to the formation of a completely insoluble gel through, presumably, unexpected cross-linking between the ylide and ester moieties [55]. 

**Scheme 3:**
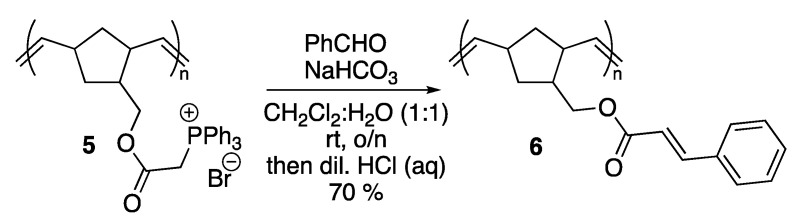
One-pot synthesis of polymeric cinnamate ester by reaction of **5** with benzaldehyde.

**Figure 1:**
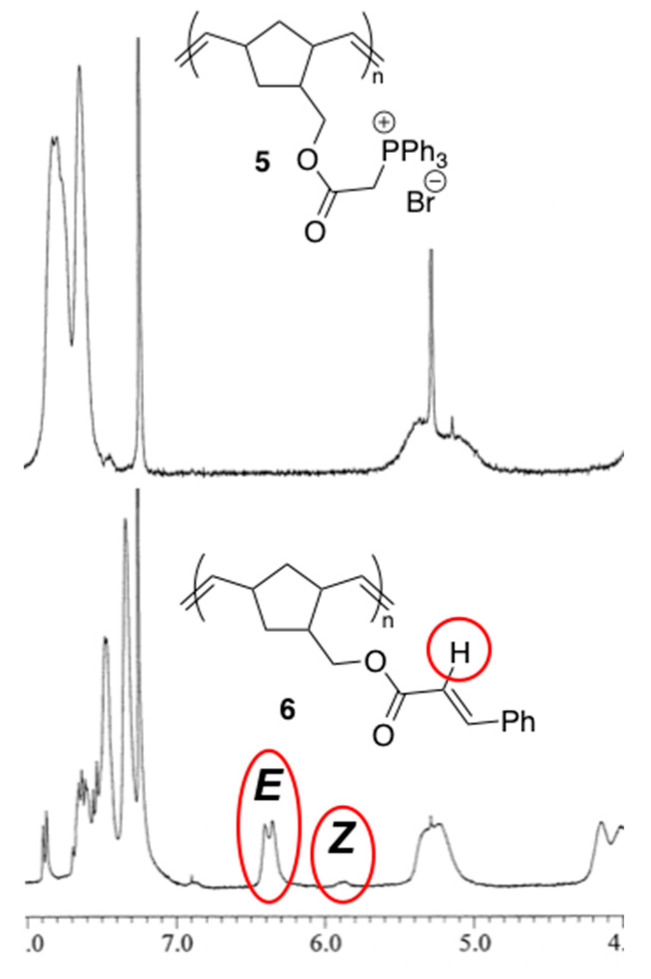
Photographs of crude, partial ^1^H NMR spectra of **5** (top) and **6** (bottom) showing the chemical shifts of the indicated protons for **6**.

We did find, however, that Wittig modifications could be carried out without the need to isolate the polymeric ylide. A biphasic, one-pot reaction of **5** (in methylene chloride), with a saturated, aqueous solution of NaHCO_3_ in the presence of excess benzaldehyde led to the formation of polymeric cinnamate ester **6** in 70% yield, after quenching with dilute acid (Scheme 3). Product formation was confirmed by ^1^H NMR spectroscopy, which showed the appearance of signals between 7.8–7.1 ppm and a doublet at 6.4 ppm, indicative of aryl and olefinic protons, respectively (Figure 1). 

We next set out to determine the aldehyde scope of this reaction. As can be seen in Table 1, aldehydes containing various electron withdrawing groups were well tolerated, providing cinnamate products in moderate to good yields with good *E/Z* ratios (Table 1, entries 1–6). Heterocyclic aromatic aldehydes (e.g., furfural) could also be utilized (Table 1, entry 7). Unfortunately, this system is not without limitations; it was discovered that aliphatic aldehydes and aromatic aldehydes containing electron donating groups resulted in substantial cross-linking, as indicated by the complete gelation of the reaction mixture (Table 1, entries 8–10). This is probably caused by the lower electrophilicity of carbonyls containing electron donating groups.

**Figure 2:**
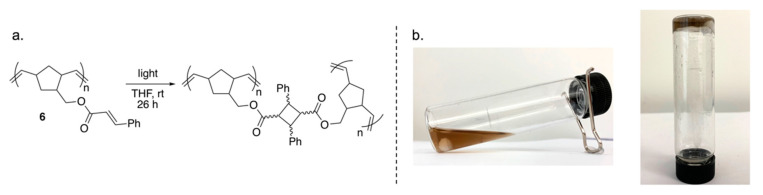
(**a**) Cross-linking of **6** through [2 + 2] cycloaddition. (**b**) Photograph of THF solution of **6** before exposure to sunlight (left) and photograph after exposure to sunlight and decantation of excess solvent (right).

Cinnamic esters (as well as other α,β-unsaturated carbonyls) have been shown to undergo synthetically useful [2 + 2] cycloaddition reactions under UV light [46]. Gratifyingly, we found that the exposure of polymer **6** (in THF) to ca. 30 h of sunlight (albeit non-continuous) was sufficient to effect the formation of a completely insoluble material that was produced, presumably, through [2 + 2] cycloaddition cross-linking reactions (Figure 2). 

## 3. Conclusions

In conclusion, we have shown that a standard Wittig reaction can be used for the post-polymerization modification of materials prepared from ROMP. Polymerization of α-bromo ester-containing monomers, followed by substitution with triphenylphosphine, led the way to carry out a one-pot reaction in which polymeric ylide formation and Wittig reaction could be carried out. This provided polymeric cinnamate esters containing electron withdrawing groups in moderate to good yield. Furthermore, these materials could be subjected to cross-linking reactions in the presence of sunlight, presumably occurring through [2 + 2] cycloadditions. Experiments designed to expand the scope of this process as well as exploring further PPM reactions utilizing Michael and Diels–Alder reactions are currently underway.

## Figures and Tables

**Table 1:** Aldehyde scope for Wittig reactions. ^1^

Entry	RCHO	Prd.	*E:Z* ^2^	Yield (%) ^3^
1	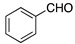	**6**	18:1	70
2	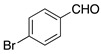	**7**	17:1	95
3	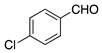	**8**	7:1	65
4	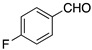	**9**	12:1	83
5	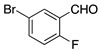	**10**	14:1	90
6	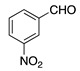	**11**	7:1	89
7	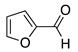	**12**	5:1	65
8	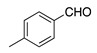	--	--	gel
9	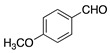	--	--	gel
10	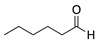	--	--	gel

^1^ Reaction conditions: [5]: [RCHO] = 1:20, saturated NaHCO_3_ (aq), CH_2_Cl_2_, room temperature, overnight. ^2^ Determined by ^1^H NMR spectroscopy. ^3^ Determined by mass of isolated product.

## Supplementary Materials

The following are available online at https://www.mdpi.com/2073-4360/12/6/1247/s1, ^1^H NMR spectra and GPC chromatograms.
