# Supplementary material for: Post-Polymerization Modification of Ring Opening Metathesis Polymerization (ROMP)-Derived Materials Using Wittig Reactions
Source: Polymers (Basel). 2020 May 29;12(6):1247. doi: 10.3390/polym12061247 (PMC7361962; doi:10.3390/polym12061247)
Supplement: Supplementary file 1 [file polymers-12-01247-s001.pdf]

Supplementary Information

Post-Polymerization Modification of ROMP-Derived Materials Using Wittig Reactions

*Ryan Duty and Christopher E. Hobbs**

Department of Chemistry, Sam Houston State University, Huntsville, Texas, USA 77340

Table of Contents

Experimental……………………………...…………………………………………....2-3
Spectral Data……………………………………...……………………….....…………..4-8
GPC Traces..……………………………………….……………….…….……………..9
References……………………………...………………………………………………..9

Experimental

General Considerations

All air or moisture sensitive reactions were carried out under a nitrogen atmosphere utilizing standard Schlenk techniques. Purification through column chromatography was accomplished with 60 Å silica gel. The elution was verified through visualization on silica gel coated glass thin layer chromatography (TLC) plates, using a 254 nm ultra-violet (UV) handheld light source for illumination. All starting materials were purchased from commercial sources (Alfa Aesar, Sigma Aldrich, and TCM Chemicals), and used without further purification, unless otherwise stated. Compounds **2** and **3** were prepared according to literature procedures.[1,2] ^{1}H and ^{13}C NMR spectroscopy was carried out on a JEOL Eclipse 300MHz spectrometer operating at 300.128 and 75.474 MHz, respectively. Chemical shifts are reported in δ (ppm) relative to the ^{1}H and ^{13}C signal of the deuterated solvent used. GPC analysis was carried out in THF at room temperature using a Viscotek TDA305 RI Detection system with a T600M general mixed column and were calculated relative to polystyrene standards.

Synthesis

*Synthesis of polymer **4***. To a 25 mL, round-bottomed flask, equipped with a magnetic stir-bar and a rubber septum was added α-bromo ester **2** (1.0 g, 4.1 mmol) and methylene chloride (10 mL). The solution was then sparged with nitrogen gas for 15 minutes. While under a positive flow of nitrogen, Grubbs 3rd generation initiator **3** (0.03 g, 0.016 mmol) was quickly added to the flask while stirring. The reaction mixture immediately turned brown upon addition of **3**. The reaction was allowed to stir for another 2 hours before being open to air and quenched with excess ethyl vinyl ether (4 mL). The polymer was isolated as a gummy solid in 80 % yield via precipitation into methanol, followed by vacuum filtration. ^{1}H NMR (300 MHz, $CDCl_3$) δ: 5.48-5.07 (m, 2H), 4.31-3.69 (m, 5H), 3.13-1.04 (m, 9H). GPC analysis: M_n = 64,500 Da, $Đ$ = 1.03.

*Synthesis of polymer **5***. To a 25 mL, round-bottomed flask, equipped with a magnetic stir-bar and a rubber septum was added polymer **4** (0.91 g, 3.69 mmol) and THF (2.6 mL). Then, triphenylphosphine (0.97 g, 3.69 mmol) was added to the reaction mixture, which was allowed to stir for 24 hours. At this point, **5** was isolated as a gummy solid in 71 % yield, via solvent precipitation into ether followed by vacuum filtration. ^{1}H NMR (300 MHz, $CDCl_3$) δ: 8.03-7.55 (m, 12H), 5.65-4.94 (m, 4H), 4.22-3.60 (m, 3H), 3.13-1.04 (m, 11H).

General procedure for Wittig reactions. To a 25 mL, round-bottomed flask, equipped with a magnetic stir-bar and a rubber septum was added polymer **5** (0.51 mmol), aldehyde (10 mmol) and methylene chloride (1 mL). To this stirring solution was added a saturated aqueous solution of $NaHCO_3$ (1.7 mL). This biphasic reaction mixture was allowed to stir overnight. At this point, the reaction was quenched by the addition HCl (0.1 M, 1.0 mL) followed by extraction with methylene chloride. The organic layer was then washed with saturated NaCl (aq) and dried over anhydrous Na_2SO_4, filtered, and the excess solvent was removed under reduced pressure. Further purification by solvent precipitation into methanol was then carried out.

Reaction with benzaldehyde. This reaction was carried out according to the general procedures above. Product was isolated in 70 % yield. ^{1}H NMR (300 MHz, $CDCl_3$) δ: 7.73-7.04 (m, 11H), 6.38 (d, 1H, J = 20 Hz), 5.45-5.04 (m, 2H), 4.28-3.79 (m, 2H), 3.14-0.77 (m, 11H).

Reaction with 4-bromobenzaldehyde. This reaction was carried out according to the general procedures above. Product was isolated in 95 % yield. ^{1}H NMR (300 MHz, $CDCl_3$) δ: 7.64-7.15 (m, 12H), 6.38 (d, 1H, J = 20 Hz), 5.45-5.04 (m, 2H), 4.28-3.79 (m, 3H), 3.14-0.77 (m, 17H).

Reaction with 4-chlorobenzaldehyde. This reaction was carried out according to the general procedures above. Product was isolated in 65 % yield. ^{1}H NMR (300 MHz, $CDCl_3$) δ: 7.76-7.30 (m, 4H), 6.45 (d, 1H, J = 18 Hz), 5.45-5.04 (m, 2H), 4.28-3.79 (m, 2H), 3.14-0.77 (m, 14H).

Reaction with 4-fluorobenzaldehyde. This reaction was carried out according to the general procedures above. Product was isolated in 83 % yield. ^{1}H NMR (300 MHz, $CDCl_3$) δ: 7.76-7.35 (m, 2H), 7.09-6.90 (m, 2H), 6.29 (d, 1H, J = 10 Hz), 5.45-5.04 (m, 2H), 4.28-3.79 (m, 2H), 3.14-0.77 (m, 10H).

Reaction with 5-bromo-2-fluorobenzaldehyde. This reaction was carried out according to the general procedures above. Product was isolated in 90 % yield. ^{1}H NMR (300 MHz, $CDCl_3$) δ: 7.71-7.17 (m, 4H), 6.35 (d, 1H, J = 20 Hz), 5.45-5.04 (m, 2H), 4.28-3.79 (m, 3H), 3.14-0.77 (m, 12H).

Reaction with 3-nitrobenzaldehyde. This reaction was carried out according to the general procedures above. Product was isolated in 89 % yield. ^{1}H NMR (300 MHz, $CDCl_3$) δ: 8.44-8.01 (m, 2H), 7.88-7.42 (m, 3H), 6.52 (d, 1H, J = 15 Hz), 5.45-5.04 (m, 2H), 4.28-3.79 (m, 2H), 3.14-0.77 (m, 14H).

Reaction with furfural. This reaction was carried out according to the general procedures above. Product was isolated in 89 % yield. ^{1}H NMR (300 MHz, $CDCl_3$) δ: 7.45 (bs, 1H), 6.56 (bs, 1H), 6.43 (bs, 1H), 6.25 (d, 1H, J = 20 Hz), 5.45-5.04 (m, 2H), 4.28-3.79 (m, 2H), 3.14-0.77 (m, 12H).

*Photo-mediated [2 + 2] crosslinking of **6***. To a 20 mL, vial, equipped with a magnetic stir bar was added polymer **6** (0.12 g) and THF (2 mL). This solution was then placed on a laboratory window sill for 3 days (ca. 30 hours of sunlight) while gently stirring. At this point, the excess solvent was decanted.

Spectral Data

^{1}H NMR Spectra

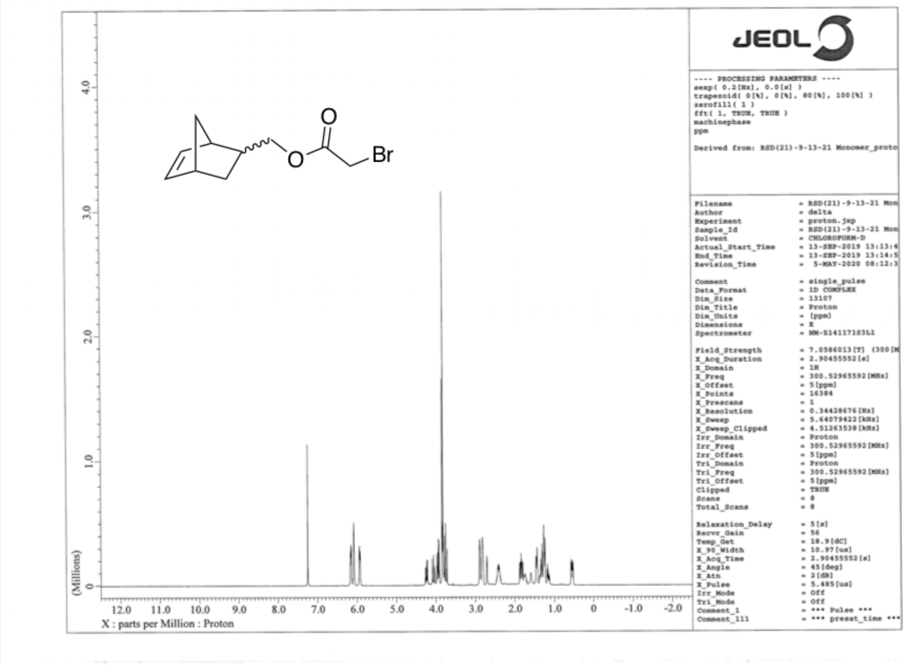

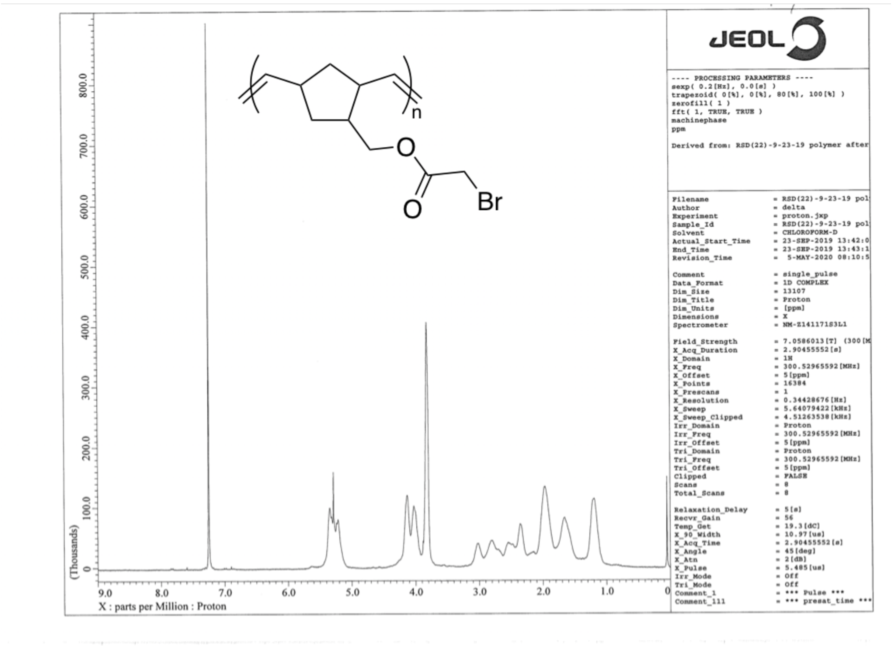

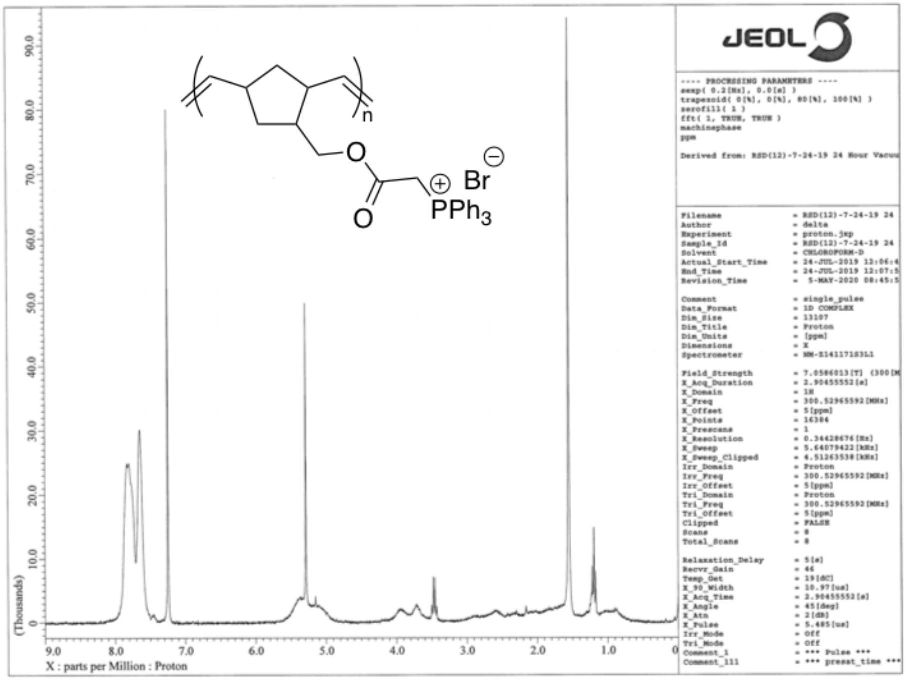
JEOL
---- PROCESSING PARAMETERS ----
sexp(0.2[Hz], 0.0[s])
trapezoid(0[%], 0[%], 80[%], 100[%])
zerofill(1)
fft(1, TRUE, TRUE)
machinephase
ppm
Derived from: RSD(12)-7-24-19 24 Hour Vacuu
Filename = RSD(12)-7-24-19 24
Author = delta
Experiment = proton.jxp
Sample_Id = RSD(12)-7-24-19 24
Solvent = CHLOROFORM-D
Actual_Start_Time = 24-JUL-2019 12:06:4
End_Time = 24-JUL-2019 12:07:5
Revision_Time = 5-MAY-2020 08:45:5
Comment = single_pulse
Data_Format = 1D COMPLEX
Dim_Size = 13107
Dim_Title = Proton
Dim_Units = [ppm]
Dimensions = X
Spectrometer = NM-Z141171S3L1
Field_Strength = 7.0586013[T] (300[M
X_Acq_Duration = 2.90455552[s]
X_Domain = 1H
X_Freq = 300.52965592[MHz]
X_Offset = 5[ppm]
X_Points = 16384
X_Prescans = 1
X_Resolution = 0.34428676[Hz]
X_Sweep = 5.64079422[kHz]
X_Sweep_Clipped = 4.51263538[kHz]
Irr_Domain = Proton
Irr_Freq = 300.52965592[MHz]
Irr_Offset = 5[ppm]
Tri_Domain = Proton
Tri_Freq = 300.52965592[MHz]
Tri_Offset = 5[ppm]
Clipped = FALSE
Scans = 8
Total_Scans = 8
Relaxation_Delay = 5[s]
Recvr_Gain = 46
Temp_Get = 19[dC]
X_90_Width = 10.97[us]
X_Acq_Time = 2.90455552[s]
X_Angle = 45[deg]
X_Atn = 2[dB]
X_Pulse = 5.485[us]
Irr_Mode = Off
Tri_Mode = Off
Comment_1 = *** Pulse ***
Comment_111 = *** presat_time ***
n
O
O
Br
PPh3
(Thousands)
X : parts per Million : Proton

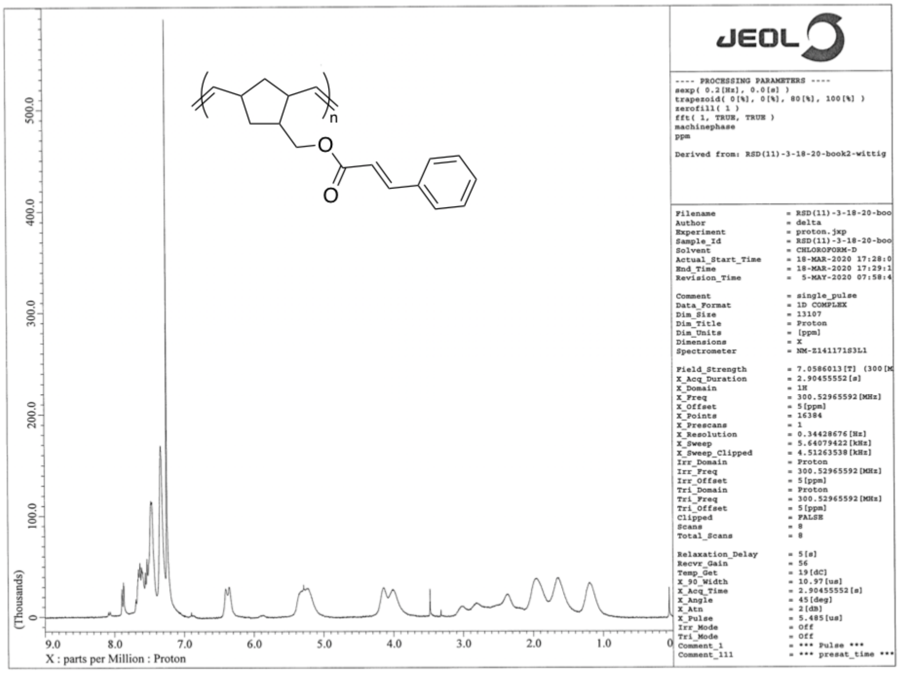
JEOL
---- PROCESSING PARAMETERS ----
sexp(0.2[Hz], 0.0[s])
trapezoid(0[%], 0[%], 80[%], 100[%])
zerofill(1)
fft(1, TRUE, TRUE)
machinephase
ppm
Derived from: RSD(11)-3-18-20-book2-wittig
Filename = RSD(11)-3-18-20-boo
Author = delta
Experiment = proton.jxp
Sample_Id = RSD(11)-3-18-20-boo
Solvent = CHLOROFORM-D
Actual_Start_Time = 18-MAR-2020 17:28:0
End_Time = 18-MAR-2020 17:29:1
Revision_Time = 5-MAY-2020 07:58:4
Comment = single_pulse
Data_Format = 1D COMPLEX
Dim_Size = 13107
Dim_Title = Proton
Dim_Units = [ppm]
Dimensions = X
Spectrometer = NM-Z141171S3L1
Field_Strength = 7.0586013[T] (300[M
X_Acq_Duration = 2.90455552[s]
X_Domain = 1H
X_Freq = 300.52965592[MHz]
X_Offset = 5[ppm]
X_Points = 16384
X_Prescans = 1
X_Resolution = 0.34428676[Hz]
X_Sweep = 5.64079422[kHz]
X_Sweep_Clipped = 4.51263538[kHz]
Irr_Domain = Proton
Irr_Freq = 300.52965592[MHz]
Irr_Offset = 5[ppm]
Tri_Domain = Proton
Tri_Freq = 300.52965592[MHz]
Tri_Offset = 5[ppm]
Clipped = FALSE
Scans = 8
Total_Scans = 8
Relaxation_Delay = 5[s]
Recvr_Gain = 56
Temp_Get = 19[dC]
X_90_Width = 10.97[us]
X_Acq_Time = 2.90455552[s]
X_Angle = 45[deg]
X_Atn = 2[dB]
X_Pulse = 5.485[us]
Irr_Mode = Off
Tri_Mode = Off
Comment_1 = *** Pulse ***
Comment_111 = *** presat_time ***
n
O
O
(Thousands)
X : parts per Million : Proton

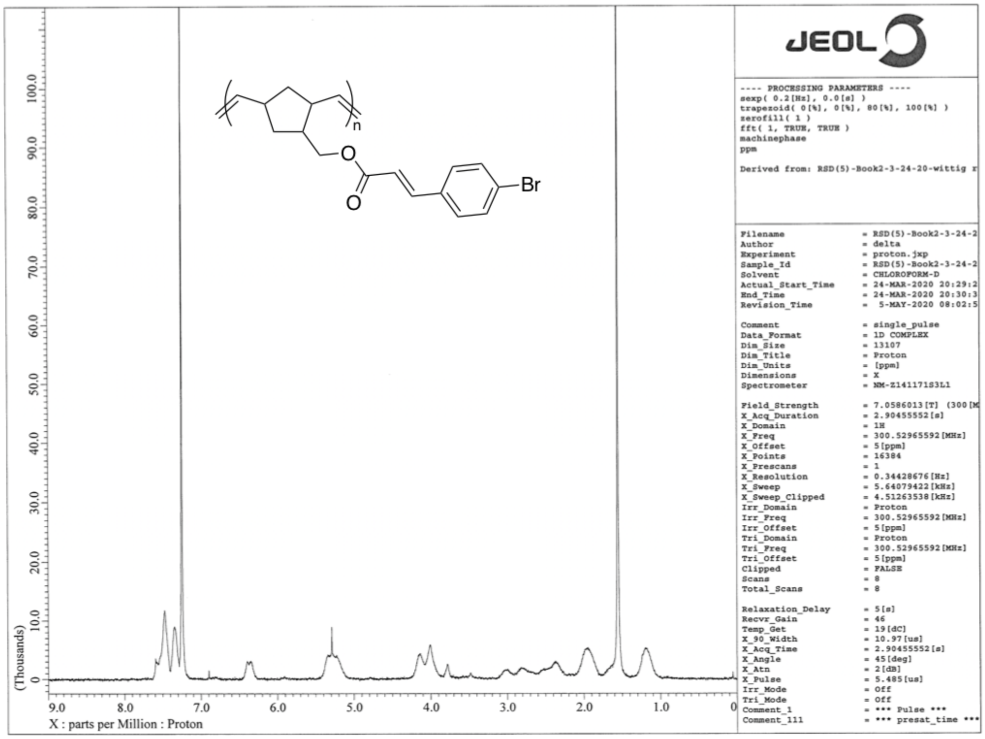
Br
n
O
O
(Thousands)
X : parts per Million : Proton
---- PROCESSING PARAMETERS ----
sexp(0.2[Hz], 0.0[s])
trapezoid(0[%], 0[%], 80[%], 100[%])
zerofill(1)
fft(1, TRUE, TRUE)
machinephase
ppm
Derived from: RSD(5)-Book2-3-24-20-wittig r
Filename = RSD(5)-Book2-3-24-2
Author = delta
Experiment = proton.jxp
Sample_Id = RSD(5)-Book2-3-24-2
Solvent = CHLOROFORM-D
Actual_Start_Time = 24-MAR-2020 20:29:2
End_Time = 24-MAR-2020 20:30:3
Revision_Time = 5-MAY-2020 08:02:5
Comment = single_pulse
Data_Format = 1D COMPLEX
Dim_Size = 13107
Dim_Title = Proton
Dim_Units = [ppm]
Dimensions = X
Spectrometer = NM-Z141171S3L1
Field_Strength = 7.0586013[T] (300[M
X_Acq_Duration = 2.90455552[s]
X_Domain = 1H
X_Freq = 300.52965592[MHz]
X_Offset = 5[ppm]
X_Points = 16384
X_Prescans = 1
X_Resolution = 0.34428676[Hz]
X_Sweep = 5.64079422[kHz]
X_Sweep_Clipped = 4.51263538[kHz]
Irr_Domain = Proton
Irr_Freq = 300.52965592[MHz]
Irr_Offset = 5[ppm]
Tri_Domain = Proton
Tri_Freq = 300.52965592[MHz]
Tri_Offset = 5[ppm]
Clipped = FALSE
Scans = 8
Total_Scans = 8
Relaxation_Delay = 5[s]
Recvr_Gain = 46
Temp_Get = 19[dC]
X_90_Width = 10.97[us]
X_Acq_Time = 2.90455552[s]
X_Angle = 45[deg]
X_Atn = 2[dB]
X_Pulse = 5.485[us]
Irr_Mode = Off
Tri_Mode = Off
Comment_1 = *** Pulse ***
Comment_111 = *** presat_time ***

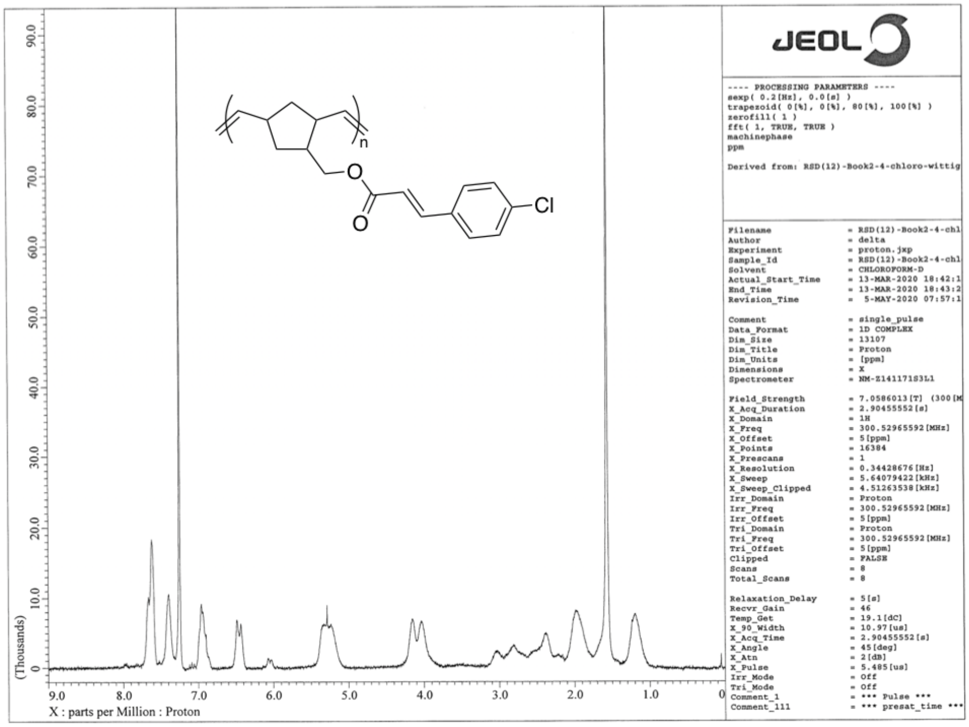
Cl
n
O
O
(Thousands)
X : parts per Million : Proton
---- PROCESSING PARAMETERS ----
sexp(0.2[Hz], 0.0[s])
trapezoid(0[%], 0[%], 80[%], 100[%])
zerofill(1)
fft(1, TRUE, TRUE)
machinephase
ppm
Derived from: RSD(12)-Book2-4-chloro-wittig
Filename = RSD(12)-Book2-4-chl
Author = delta
Experiment = proton.jxp
Sample_Id = RSD(12)-Book2-4-chl
Solvent = CHLOROFORM-D
Actual_Start_Time = 13-MAR-2020 18:42:1
End_Time = 13-MAR-2020 18:43:2
Revision_Time = 5-MAY-2020 07:57:1
Comment = single_pulse
Data_Format = 1D COMPLEX
Dim_Size = 13107
Dim_Title = Proton
Dim_Units = [ppm]
Dimensions = X
Spectrometer = NM-Z141171S3L1
Field_Strength = 7.0586013[T] (300[M
X_Acq_Duration = 2.90455552[s]
X_Domain = 1H
X_Freq = 300.52965592[MHz]
X_Offset = 5[ppm]
X_Points = 16384
X_Prescans = 1
X_Resolution = 0.34428676[Hz]
X_Sweep = 5.64079422[kHz]
X_Sweep_Clipped = 4.51263538[kHz]
Irr_Domain = Proton
Irr_Freq = 300.52965592[MHz]
Irr_Offset = 5[ppm]
Tri_Domain = Proton
Tri_Freq = 300.52965592[MHz]
Tri_Offset = 5[ppm]
Clipped = FALSE
Scans = 8
Total_Scans = 8
Relaxation_Delay = 5[s]
Recvr_Gain = 46
Temp_Get = 19.1[dC]
X_90_Width = 10.97[us]
X_Acq_Time = 2.90455552[s]
X_Angle = 45[deg]
X_Atn = 2[dB]
X_Pulse = 5.485[us]
Irr_Mode = Off
Tri_Mode = Off
Comment_1 = *** Pulse ***
Comment_111 = *** presat_time ***

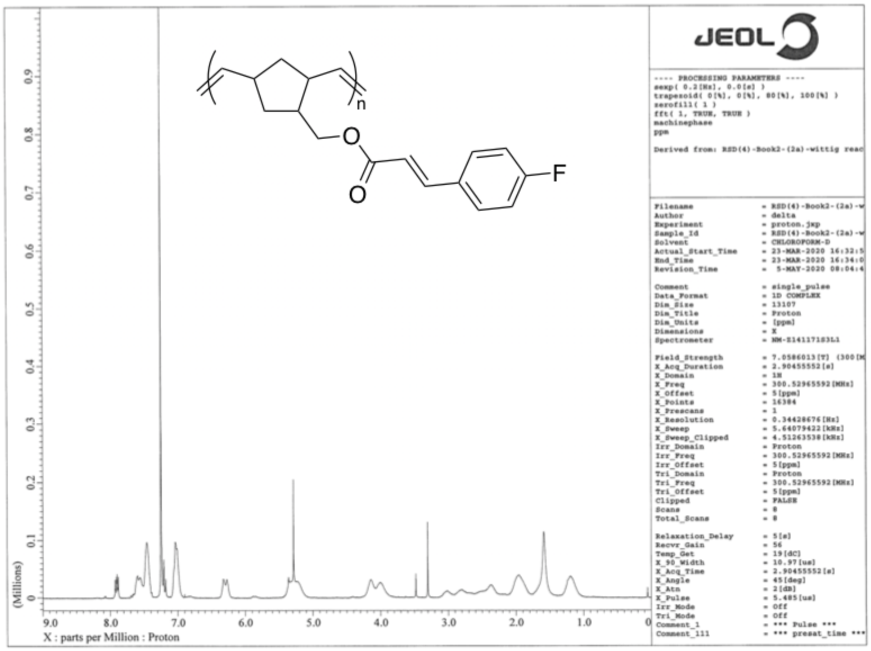
n
O
O
F
(Millions)
0
0.1
0.2
0.3
0.4
0.5
0.6
0.7
0.8
0.9
9.0
8.0
7.0
6.0
5.0
4.0
3.0
2.0
1.0
0
X : parts per Million : Proton
---- PROCESSING PARAMETERS ----
sexp(0.2[Hz], 0.0[s])
trapezoid(0[%], 0[%], 80[%], 100[%])
zerofill(1)
fft(1, TRUE, TRUE)
machinephase
ppm
Derived from: RSD(4)-Book2-(2a)-wittig reac
Filename = RSD(4)-Book2-(2a)-w
Author = delta
Experiment = proton.jxp
Sample_Id = RSD(4)-Book2-(2a)-w
Solvent = CHLOROFORM-D
Actual_Start_Time = 23-MAR-2020 16:32:5
End_Time = 23-MAR-2020 16:34:0
Revision_Time = 5-MAY-2020 08:04:4
Comment = single_pulse
Data_Format = 1D COMPLEX
Dim_Size = 13107
Dim_Title = Proton
Dim_Units = [ppm]
Dimensions = X
Spectrometer = NM-Z14117183L1
Field_Strength = 7.0586013[T] (300[M
X_Acq_Duration = 2.90455552[s]
X_Domain = 1H
X_Freq = 300.52965592[MHz]
X_Offset = 5[ppm]
X_Points = 16384
X_Prescans = 1
X_Resolution = 0.34428676[Hz]
X_Sweep = 5.64079422[kHz]
X_Sweep_Clipped = 4.51263538[kHz]
Irr_Domain = Proton
Irr_Freq = 300.52965592[MHz]
Irr_Offset = 5[ppm]
Tri_Domain = Proton
Tri_Freq = 300.52965592[MHz]
Tri_Offset = 5[ppm]
Clipped = FALSE
Scans = 8
Total_Scans = 8
Relaxation_Delay = 5[s]
Recvr_Gain = 56
Temp_Get = 19[dC]
X_90_Width = 10.97[us]
X_Acq_Time = 2.90455552[s]
X_Angle = 45[deg]
X_Atn = 2[dB]
X_Pulse = 5.485[us]
Irr_Mode = Off
Tri_Mode = Off
Comment_1 = *** Pulse ***
Comment_111 = *** presat_time ***

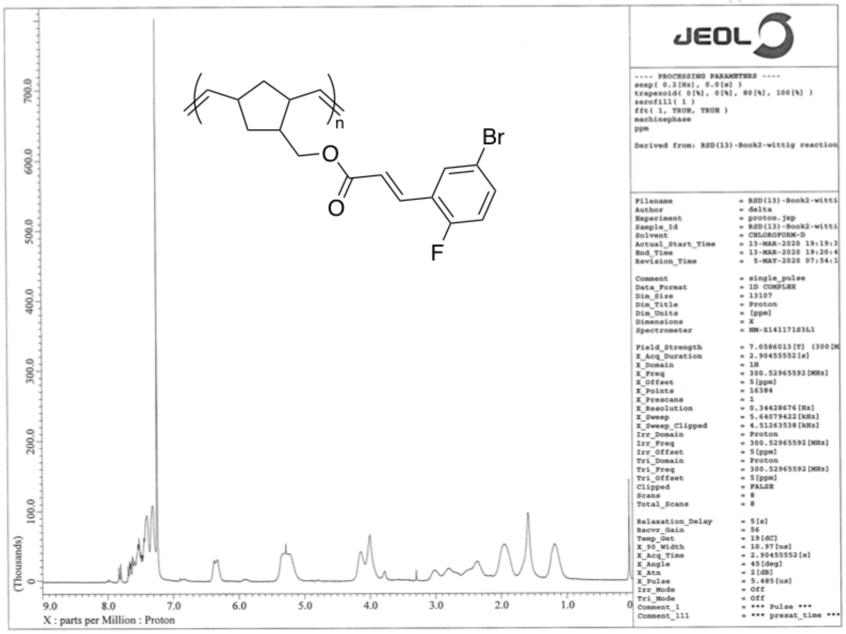
n
O
O
Br
F
(Thousands)
0
100.0
200.0
300.0
400.0
500.0
600.0
700.0
9.0
8.0
7.0
6.0
5.0
4.0
3.0
2.0
1.0
0
X : parts per Million : Proton
---- PROCESSING PARAMETERS ----
sexp(0.2[Hz], 0.0[s])
trapezoid(0[%], 0[%], 80[%], 100[%])
zerofill(1)
fft(1, TRUE, TRUE)
machinephase
ppm
Derived from: RSD(13)-Book2-wittig reaction
Filename = RSD(13)-Book2-witti
Author = delta
Experiment = proton.jxp
Sample_Id = RSD(13)-Book2-witti
Solvent = CHLOROFORM-D
Actual_Start_Time = 13-MAR-2020 19:19:3
End_Time = 13-MAR-2020 19:20:4
Revision_Time = 5-MAY-2020 07:54:1
Comment = single_pulse
Data_Format = 1D COMPLEX
Dim_Size = 13107
Dim_Title = Proton
Dim_Units = [ppm]
Dimensions = X
Spectrometer = NM-Z14117183L1
Field_Strength = 7.0586013[T] (300[M
X_Acq_Duration = 2.90455552[s]
X_Domain = 1H
X_Freq = 300.52965592[MHz]
X_Offset = 5[ppm]
X_Points = 16384
X_Prescans = 1
X_Resolution = 0.34428676[Hz]
X_Sweep = 5.64079422[kHz]
X_Sweep_Clipped = 4.51263538[kHz]
Irr_Domain = Proton
Irr_Freq = 300.52965592[MHz]
Irr_Offset = 5[ppm]
Tri_Domain = Proton
Tri_Freq = 300.52965592[MHz]
Tri_Offset = 5[ppm]
Clipped = FALSE
Scans = 8
Total_Scans = 8
Relaxation_Delay = 5[s]
Recvr_Gain = 56
Temp_Get = 19[dC]
X_90_Width = 10.97[us]
X_Acq_Time = 2.90455552[s]
X_Angle = 45[deg]
X_Atn = 2[dB]
X_Pulse = 5.485[us]
Irr_Mode = Off
Tri_Mode = Off
Comment_1 = *** Pulse ***
Comment_111 = *** presat_time ***

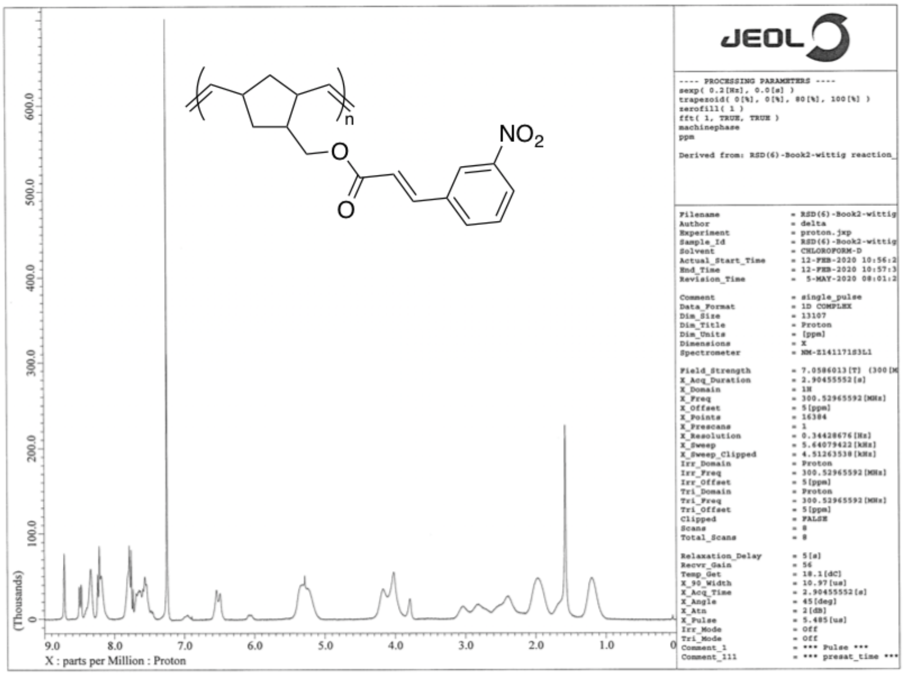
NO2
O
O
n
JEOL
---- PROCESSING PARAMETERS ----
sexp(0.2[Hz], 0.0[s])
trapezoid(0[%], 0[%], 80[%], 100[%])
zerofill(1)
fft(1, TRUE, TRUE)
machinephase
ppm
Derived from: RSD(6)-Book2-wittig reaction_
Filename = RSD(6)-Book2-wittig
Author = delta
Experiment = proton.jxp
Sample_Id = RSD(6)-Book2-wittig
Solvent = CHLOROFORM-D
Actual_Start_Time = 12-FEB-2020 10:56:2
End_Time = 12-FEB-2020 10:57:3
Revision_Time = 5-MAY-2020 08:01:2
Comment = single_pulse
Data_Format = 1D COMPLEX
Dim_Size = 13107
Dim_Title = Proton
Dim_Units = [ppm]
Dimensions = X
Spectrometer = NM-Z141171S3L1
Field_Strength = 7.0586013[T] (300[M
X_Acq_Duration = 2.90455552[s]
X_Domain = 1H
X_Freq = 300.52965592[MHz]
X_Offset = 5[ppm]
X_Points = 16384
X_Prescans = 1
X_Resolution = 0.34428676[Hz]
X_Sweep = 5.64079422[kHz]
X_Sweep_Clipped = 4.51263538[kHz]
Irr_Domain = Proton
Irr_Freq = 300.52965592[MHz]
Irr_Offset = 5[ppm]
Tri_Domain = Proton
Tri_Freq = 300.52965592[MHz]
Tri_Offset = 5[ppm]
Clipped = FALSE
Scans = 8
Total_Scans = 8
Relaxation_Delay = 5[s]
Recvr_Gain = 56
Temp_Get = 18.1[dC]
X_90_Width = 10.97[us]
X_Acq_Time = 2.90455552[s]
X_Angle = 45[deg]
X_Atn = 2[dB]
X_Pulse = 5.485[us]
Irr_Mode = Off
Tri_Mode = Off
Comment_1 = *** Pulse ***
Comment_111 = *** presat_time ***
(Thousands)
0
100.0
200.0
300.0
400.0
500.0
600.0
9.0
8.0
7.0
6.0
5.0
4.0
3.0
2.0
1.0
0
X : parts per Million : Proton

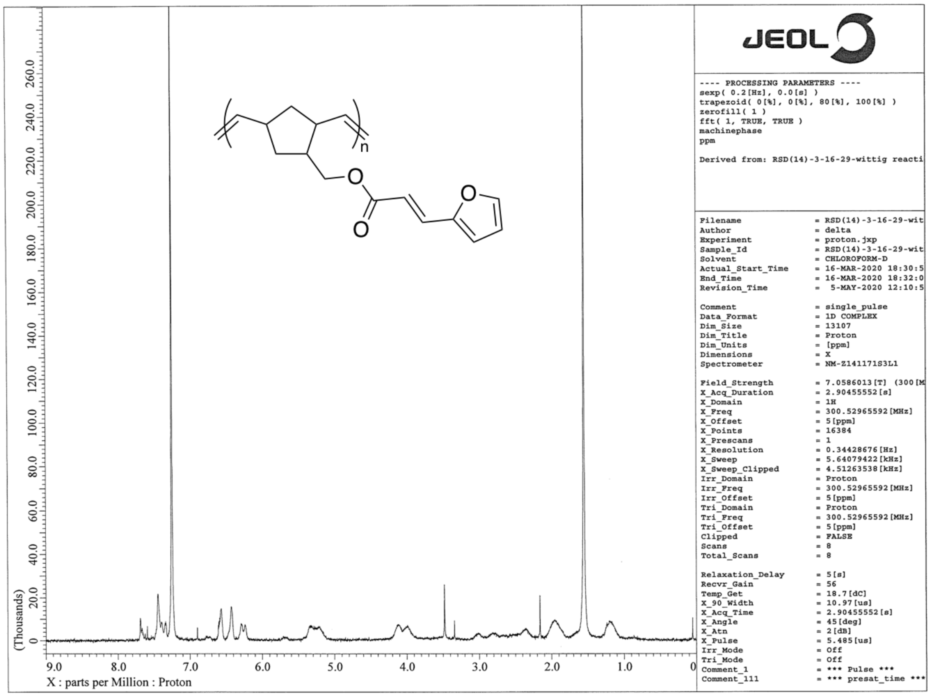
O
O
O
n
JEOL
---- PROCESSING PARAMETERS ----
sexp(0.2[Hz], 0.0[s])
trapezoid(0[%], 0[%], 80[%], 100[%])
zerofill(1)
fft(1, TRUE, TRUE)
machinephase
ppm
Derived from: RSD(14)-3-16-29-wittig reacti
Filename = RSD(14)-3-16-29-wit
Author = delta
Experiment = proton.jxp
Sample_Id = RSD(14)-3-16-29-wit
Solvent = CHLOROFORM-D
Actual_Start_Time = 16-MAR-2020 18:30:5
End_Time = 16-MAR-2020 18:32:0
Revision_Time = 5-MAY-2020 12:10:5
Comment = single_pulse
Data_Format = 1D COMPLEX
Dim_Size = 13107
Dim_Title = Proton
Dim_Units = [ppm]
Dimensions = X
Spectrometer = NM-Z141171S3L1
Field_Strength = 7.0586013[T] (300[M
X_Acq_Duration = 2.90455552[s]
X_Domain = 1H
X_Freq = 300.52965592[MHz]
X_Offset = 5[ppm]
X_Points = 16384
X_Prescans = 1
X_Resolution = 0.34428676[Hz]
X_Sweep = 5.64079422[kHz]
X_Sweep_Clipped = 4.51263538[kHz]
Irr_Domain = Proton
Irr_Freq = 300.52965592[MHz]
Irr_Offset = 5[ppm]
Tri_Domain = Proton
Tri_Freq = 300.52965592[MHz]
Tri_Offset = 5[ppm]
Clipped = FALSE
Scans = 8
Total_Scans = 8
Relaxation_Delay = 5[s]
Recvr_Gain = 56
Temp_Get = 18.7[dC]
X_90_Width = 10.97[us]
X_Acq_Time = 2.90455552[s]
X_Angle = 45[deg]
X_Atn = 2[dB]
X_Pulse = 5.485[us]
Irr_Mode = Off
Tri_Mode = Off
Comment_1 = *** Pulse ***
Comment_111 = *** presat_time ***
(Thousands)
0
20.0
40.0
60.0
80.0
100.0
120.0
140.0
160.0
180.0
200.0
220.0
240.0
260.0
9.0
8.0
7.0
6.0
5.0
4.0
3.0
2.0
1.0
0
X : parts per Million : Proton

GPC Trace

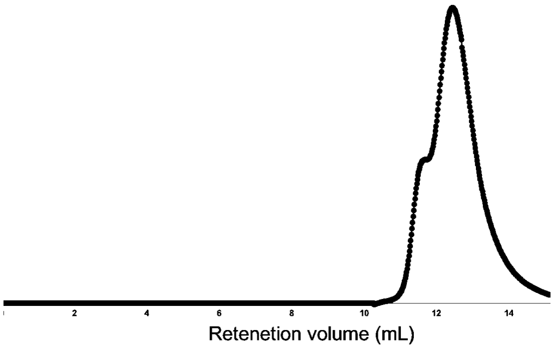

References

1. Taylor, M.T.; Nelson, J.E.; Suero, M.G.; Gaunt, M.J. A protein functionalization platform based on selective reactions at methionine residues. *Nature* **2018**, *562*, 563–568, doi:10.1038/s41586-018-0608-y.
2. Love, J.A.; Morgan, J.P.; Trnka, T.M.; Grubbs, R.H. A Practical and Highly Active Ruthenium-Based Catalyst that Effects the Cross Metathesis of Acrylonitrile. *Angew. Chemie Int. Ed.* **2002**, *41*, 4035–4037, doi:10.1002/1521-3773(20021104)41:21<4035::AID-ANIE4035>3.0.CO;2-I.
